# Proteomic biomarkers and biological pathways associated with atrial fibrillation in heart failure patients with reduced ejection fraction

**DOI:** 10.1016/j.hroo.2025.06.007

**Published:** 2025-06-16

**Authors:** Teun B. Petersen, Mylène Barry-Loncq de Jong, Jie Fen Chin, Navin Suthahar, Peter J. van der Spek, Peter D. Katsikis, K. Martijn Akkerhuis, Victor A. Umans, Rudolf A. de Boer, Bas M. van Dalen, Jasper J. Brugts, Folkert W. Asselbergs, Eric Boersma, Dimitris Rizopoulos, Sing-Chien Yap, Isabella Kardys

**Affiliations:** 1 Department of Cardiology, Thorax Center, Cardiovascular Institute, Erasmus MC, Rotterdam, The Netherlands; 2 Department of Biostatistics, Erasmus MC, Rotterdam, The Netherlands; 3 Department of Epidemiology, Erasmus MC, Rotterdam, The Netherlands; 4 Department of Cardiology, Franciscus Gasthuis & Vlietland, Rotterdam, The Netherlands; 5 Department of Pathology & Clinical Bioinformatics, Erasmus MC, Rotterdam, The Netherlands; 6 Department of Immunology, Erasmus MC, Rotterdam, The Netherlands; 7 Department of Cardiology, Northwest Clinics, Alkmaar, The Netherlands; 8 Department of Cardiology, Amsterdam University Medical Centers, University of Amsterdam, Amsterdam, The Netherlands

**Keywords:** Heart failure, HFrEF, Atrial fibrillation, Proteomics, Phenotype

## Abstract

**Background:**

Atrial fibrillation (AF) and heart failure (HF) are intertwined conditions with high mortality and impact on quality of life. The biological mechanisms at play in patients with HF with vs without AF may differ.

**Objectives:**

This study aimed to describe differences in circulating proteins with putative pathophysiological effects between HF with reduced ejection fraction (HFrEF) patients with and without AF.

**Methods:**

We examined 377 patients with ambulant HFrEF and measured 4210 circulating proteins in baseline blood samples using an aptamer-based multiplex proteomic approach. Associations between AF (AF history or on baseline electrocardiogram) and the proteins were assessed using regression models adjusted for age, sex, kidney function, and duration of HF at baseline. Associations of AF-related proteins with biological processes were evaluated using enrichment analyses.

**Results:**

The median age [25th–75th percentile] was 64 years [55–72], 73% [274 of 377] were male, 28% [104 of 375] had New York Heart Association class III/IV, and 37% [139 of 377] had AF (either AF history [36%, 137 of 377] or AF on baseline electrocardiogram [8%, 30 of 374]). We found 71 proteins significantly associated with AF (false discovery rate < .05), including well-studied (eg, troponin T, insulin-like growth factor-binding protein 7, microfibril-associated glycoprotein 4, bone morphogenetic protein 10, angiopoietin 2) and lesser-studied proteins (eg, olfactomedin−like protein 3, keratocan, basigin) in the AF domain. Our pathway analysis revealed modules of proteins related to various underlying mechanisms, such as nervous system development, elastic fiber assembly, protein glycosylation, and ether lipid metabolism.

**Conclusions:**

Patients with HFrEF with AF have distinct circulating proteomic profiles, and these differences are related to various biological mechanisms. This study provides an overview of the systemic biological pathways associated with AF in patients with HFrEF, confirms (pre-)clinical findings regarding AF-related proteins, and could inform future research in novel treatment targets and HFrEF-AF management after careful validation.

## Graphical Abstract

**Figure:**
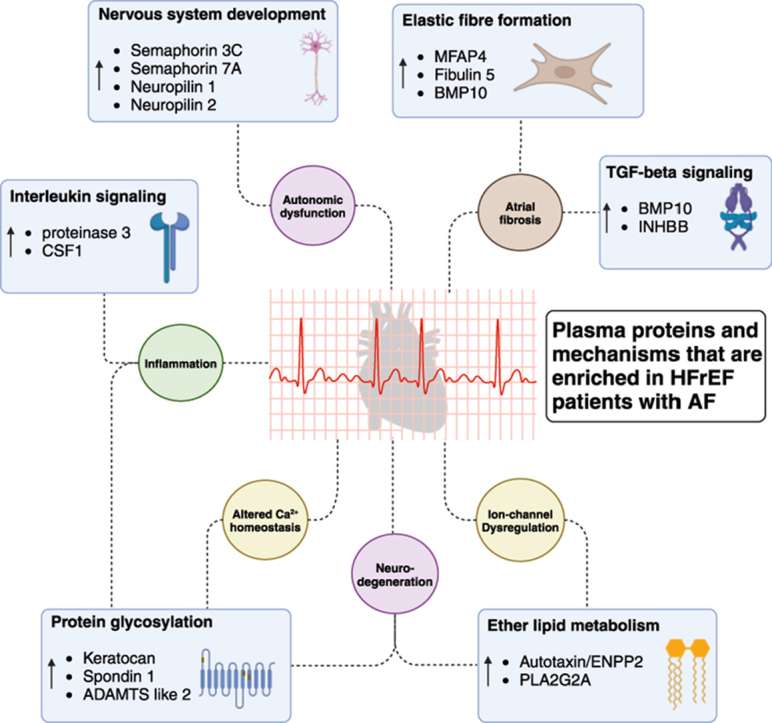

**Key Findings**

▪ Atrial fibrillation (AF) and heart failure (HF) are prevalent comorbidities that have a negative impact on quality of life and mortality. The underlying pathophysiology of HF may differ between patients with and without AF.
▪ Patients with HF with reduced ejection fraction (HFrEF) and concomitant AF have distinct plasma proteomic profiles compared with patients with HFrEF without AF.
▪ These proteomic profiles are related to various biological mechanisms implicated in the pathophysiology of HF, such as elastic fiber assembly, nervous system development, protein glycosylation, and ether lipid metabolism.

## Introduction

Atrial fibrillation (AF) and heart failure (HF) are intertwined conditions with high mortality rates and a strong impact on quality of life. Their coexistence is increasingly prevalent, with both diseases showing rising incidence rates in recent years.^1^ AF and HF are known to have a reciprocal influence on their development and progression and share many risk factors, such as hypertension, obesity, diabetes mellitus, and ischemic heart disease. Their combined presence further increases the risk of hospitalization and mortality.^2^, ^3^, ^4^, ^5^ Thus, the pathophysiology of HF may differ between patients with and without AF.

Identifying differences in levels of plasma proteins with putative pathophysiological effects in HF subgroups, such as the HF-AF phenotype, is crucial for developing effective strategies for HF management, given that such subgroups may respond differently to treatments.^6^ Previous studies have linked AF and HF through mechanisms such as cardiac remodeling, neurohormonal activation, and rate-related left ventricular impairment.^3^^,^^7^

In this study, we investigated the intricate relationship between AF and HF by exploring biological pathways associated with AF in a population of patients with HF with reduced ejection fraction (HFrEF) and identifying associated plasma proteins from an elaborate panel of 4210 proteins.

## Methods

### Study population and study design

The current investigation was performed in the Serial Biomarker Measurements and New Echocardiographic Techniques in Chronic Heart Failure Patients Result in Tailored Prediction of Prognosis study, a prospective cohort study of patients with stable chronic HF (CHF) conducted in the Erasmus MC, Rotterdam, and Northwest Clinics, Alkmaar, The Netherlands.^8^ Patients were recruited during regular outpatient visits and included if they were 18 years or older, were able to understand and sign the informed consent form, were diagnosed as having CHF ≥3 months ago according to the guidelines of the European Society of Cardiology,^9^^,^^10^ and had not been hospitalized for HF less than 3 months before inclusion. Left ventricular ejection fraction (LVEF) was extracted from the hospital records to assess suitability for inclusion. During the study, information about the patients was recorded at baseline and at predefined follow-up visits, which were scheduled every 3 months (±1). These visits included the collection of blood samples, short medical examinations, and documentation of adverse cardiovascular (CV) events since the last visit. A total of 398 patients with CHF were enrolled between August 2011 and January 2018. The current investigation concerns the 377 patients with HFrEF and known AF status. The study was approved by the medical ethics committee of the Erasmus MC in Rotterdam and complied with the Declaration of Helsinki. All included patients provided a written informed consent.

### Clinical assessment at baseline

At baseline, all patients were evaluated by a research physician or a research nurse. This evaluation included documentation of HF-related symptoms, New York Heart Association (NYHA) class, and a physical examination. Medical history, HF etiology, and medication use were extracted from hospital records. An electrocardiogram (ECG) was conducted at baseline, as was echocardiography. For echocardiography, 2-dimensional grayscale harmonic images were obtained in the left lateral decubitus position using a commercially available ultrasound system (iE33, Philips, Best, The Netherlands), equipped with a broadband (1–5 MHz) S5-1 transducer (frequency transmitted 1.7 MHz, received 3.4 MHz), and stored in the echo core laboratory of Erasmus MC. Using specialized software (TomTec Imaging Systems, Unterschleissheim, Germany), parameters including LVEF were measured. LVEF was measured using the 2-dimensional triplane method.

### Outcome definitions

AF status was assessed at baseline. For the main analysis, patients were considered to have AF at baseline if they had a documented history of AF in their hospital records or when AF was registered on the standard ECG conducted at baseline.

The study endpoints during follow-up were determined by a clinical event committee based on hospital records, discharge letters, and event definitions, without knowledge of the proteomic measurements. In patients with multiple endpoints, only the first was considered for analysis. The clinical primary endpoint (PEP) was predefined as the composite of CV death, left ventricular assist device implantation, heart transplantation, and hospitalization for acute or worsened HF. Patients were considered hospitalized for acute or worsened HF when hospitalized for an exacerbation of HF symptoms, together with 2 of the following conditions: N-terminal pro–B-type natriuretic peptide (NT-proBNP) or B-type natriuretic peptide exceeding 3 times the normal upper limit; signs of worsening HF, such as pulmonary rales, raised jugular venous pressure, or peripheral edema; increased dose or intravenous administration of diuretics; or administration of positive inotropic agents.

### Proteomic measurements

Blood samples used for the current investigation were collected at baseline, processed within 2 hours after collection, and subsequently stored at −80°C. The proteomic analyses were performed on 55 μL of EDTA plasma, using the aptamer-based proteomic SomaScan platform.^11^ In total, 5284 aptamers were applied in the samples. We excluded 300 aptamers with nonvalidated or nonhuman targets. Furthermore, when aptamers targeted the same protein, we kept the aptamer with the highest binding affinity and excluded the others. These steps left us with 4210 aptamers corresponding to an equal number of proteins. Additional information is presented in the Supplemental Methods and Supplemental Table 1.

### Statistical analysis

Differences in the clinical characteristics at baseline between patients with HFrEF with and without AF were assessed using Wilcoxon rank-sum tests, χ^2^ tests, or Fisher’s exact tests where appropriate. Differences in the hazard of the PEP between these groups were investigated through Kaplan-Meier curves and a log-rank test. The proteins were log transformed and standardized to *z-*scores before all further analyses. Associations between AF and baseline protein levels were determined using single-protein linear regression models, with the protein as the outcome variable and AF status as the exposure variable. The models were adjusted for age, sex, kidney function (eGFR CKD-EPI), and HF duration. Proteins significantly associated with AF (false discovery rate [FDR] <0.05) were selected for further analysis. A sensitivity analysis was conducted to assess the potential confounding effect of patient characteristics and comorbidities, including diabetes mellitus, hypertension, hypercholesterolemia, myocardial infarction, NYHA class, body mass index, smoking status, and use of medications that showed significant differences between patients with AF and without AF.

Proteins associated with AF were connected in a knowledge-based network using the STRING database, a database of protein-protein associations, where modules of closely associated proteins could be determined using optimal modularity network clustering.^12^^,^^13^ Subsequently, these modules were analyzed for their associations with pathways of the Kyoto Encyclopedia of Genes and Genomes (KEGG) and the Reactome Pathway Knowledge base by separate enrichment analyses conducted using the ToppGene suite, with the full set of 4210 proteins taken as reference.^14^, ^15^, ^16^ This method of preclustering the set of proteins has been shown to provide more profound insights into associated biological pathways when multiple are present, without raising the number of false positive pathways.^17^

R version 4.4.1 was used for all analyses. Two-sided *P-*values of <.05 were considered statistically significant for the analyses concerning baseline characteristics and FDR of <.05 for the protein analyses.

## Results

### Differences in baseline characteristics and prognosis

Baseline characteristics are presented in Table 1. Median age (25th–75th percentile) of the study patients was 64 years [55–72], 73% were men, and 37% had AF (either AF history [36%] or AF on baseline ECG [8%]). Patients with AF were older (69 [62–77] vs 61 [52–69] years) and more often men (81% vs 68%). Their duration of HF at inclusion was longer (5.2 [2.6–9.7] vs 3.6 [1.2–9.6] years), and their baseline levels of NT-proBNP (1649 [837–3205] vs 803 [245–1894] ng/L) and high-sensitivity troponin T (25 [14–42] vs 14 [8–24] ng/L) were higher. Prevalence of cardiomyopathy was similar; however, patients with AF were more likely to have hypertrophic (7.2% vs 2.1%) rather than dilated cardiomyopathy (19% vs 30%). Hypertensive etiology (13% vs 5.9%) and etiology secondary to valvular heart disease (6.5% vs 1.3%), as well as chronic renal failure (60% vs 41%), were more prevalent in those with AF. The use of anticoagulants (90% vs 63%) and loop diuretics (96% vs 90%) was more prevalent in patients with AF, whereas the use of angiotensin-converting enzyme inhibitors (59% vs 73%) and aspirin (6.5% vs 29%) was less prevalent.

**Table 1:** Baseline characteristics

Characteristic	Total, N = 377∗	Atrial fibrillation, n = 139∗	No atrial fibrillation, n = 238∗	*P*-value†
Demographics				
Age at baseline visit (y)	64 (55–72)	69 (62–77)	61 (52–69)	**<.001**
Men	274 (73)	112 (81)	162 (68)	**.009**
Ethnicity: Caucasian	346 (93)	132 (96)	214 (91)	.078
Features of HF				
Duration at baseline (y)	4.4 (1.6–9.7)	5.2 (2.6–9.7)	3.6 (1.2–9.6)	**.008**
NYHA class				**.047**
I	93 (25)	31 (22)	62 (26)	
II	178 (47)	61 (44)	117 (50)	
III	101 (27)	44 (32)	57 (24)	
IV	3 (0.8)	3 (2.2)	0 (0)	
Systolic ejection fraction (%)	30 (23–36)	30 (24–40)	29 (22–35)	.13
Clinical characteristics				
BMI (kg/m^2^)	26.5 (24.0–30.1)	26.2 (24.3–29.3)	26.7 (23.7–30.3)	>.9
eGFR CKD-EPI (mL/min/1.73 m^2^)	57 (43–74)	53 (40–69)	59 (44–76)	**.030**
Systolic blood pressure (mm Hg)	114 (100–128)	116 (100–128)	114 (100–130)	.7
Diastolic blood pressure (mm Hg)	70 (60–78)	70 (60–77)	70 (60–78)	.9
Biomarker level				
NT-proBNP				**<.001**
pmol/L	126 (45–273)	195 (99–379)	95 (29–224)	
ng/L	1066 (381–2308)	1649 (837–3205)	803 (245–1894)	
hs-troponin T (ng/L)	18 (9–33)	25 (14–42)	14 (8–24)	**<.001**
CRP (mg/L)	2.2 (0.9–4.9)	2.3 (1.1–5.3)	2.0 (0.8–4.3)	.2
Etiology of HF				
Ischemic heart disease	164 (44)	57 (41)	107 (45)	.5
Hypertension	32 (8.5)	18 (13)	14 (5.9)	**.018**
Secondary to valvular heart disease	12 (3.2)	9 (6.5)	3 (1.3)	**.011**
Cardiomyopathy	122 (32)	41 (29)	81 (34)	.4
Hypertrophic (HCM)	15 (4.0)	10 (7.2)	5 (2.1)	**.015**
Dilated (DCM)	97 (26)	26 (19)	71 (30)	**.017**
Restrictive	0 (0)	0 (0)	0 (0)	>.9
Arrhythmogenic right ventricular cardiomyopathy	1 (0.3)	0 (0)	1 (0.4)	>.9
Noncompaction cardiomyopathy	4 (1.1)	2 (1.4)	2 (0.8)	.6
Unclassified	7 (1.9)	4 (2.9)	3 (1.3)	.4
Unknown	25 (6.6)	7 (5.0)	18 (7.6)	.3
Medical history				
Myocardial infarction	144 (39)	53 (38)	91 (39)	>.9
PCI	125 (33)	41 (29)	84 (35)	.2
CABG	54 (14)	26 (19)	28 (12)	.063
Stroke (CVA/TIA)	47 (13)	18 (13)	29 (12)	.8
Chronic renal failure	179 (48)	83 (60)	96 (41)	**<.001**
Diabetes mellitus	98 (26)	42 (30)	56 (24)	.2
Known hypercholesterolemia	159 (43)	65 (47)	94 (41)	.2
Hypertension	164 (44)	69 (50)	95 (40)	.067
Intoxication				
Smoking: ever	269 (72)	104 (75)	165 (70)	.2
Smoking: current	37 (9.9)	13 (9.4)	24 (10)	.8
Medication				
Ace inhibitor	255 (68)	82 (59)	173 (73)	**.008**
Angiotensin II receptor blockers	105 (28)	46 (33)	59 (25)	.083
Aldosterone antagonists	288 (76)	109 (78)	179 (75)	.5
Loop diuretics	348 (92)	134 (96)	214 (90)	**.023**
Thiazide diuretics	12 (3.2)	6 (4.3)	6 (2.5)	.4
Other diuretics	5 (1.3)	2 (1.5)	3 (1.3)	>.9
Beta blockers	345 (92)	123 (88)	222 (94)	.078
Aspirin	77 (20)	9 (6.5)	68 (29)	**<.001**
Anticoagulants	274 (73)	125 (90)	149 (63)	**<.001**

BMI = body mass index; CABG = coronary artery bypass graft; CRP = C-reactive protein; CVA = cerebrovascular accident; DCM = dilated cardiomyopathy; eGFR = estimated glomerular filtration rate; HCM = hypertrophic cardiomyopathy; HF = heart failure; hs = high-sensitivity; NYHA = New York Heart Association; PCI = percutaneous coronary intervention; TIA = transient ischemic attack.

∗ Median (interquartile range); n (%).

† Wilcoxon rank-sum test; Pearson’s χ^2^ test; Fisher’s exact test; *P*-value < .05.

**Figure 1:**
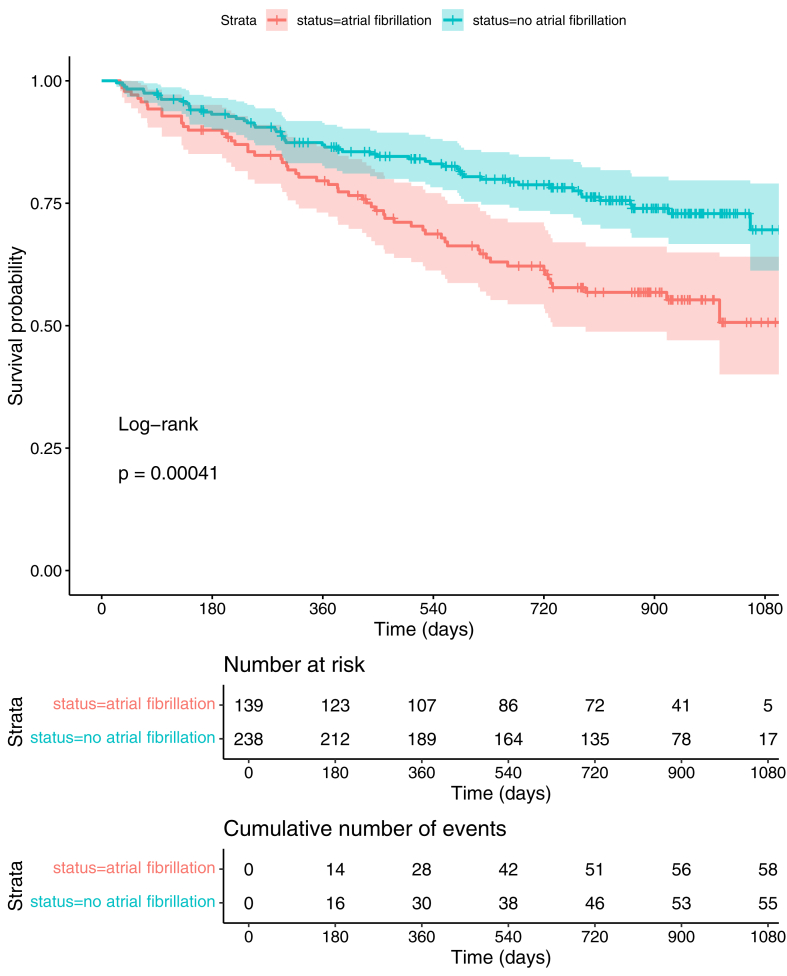
Differences in prognosis for patients with HFrEF with and without AF. Kaplan-Meier plot illustrating the differences in prognosis for patients with HFrEF with and without AF. AF = atrial fibrillation; HFrEF = heart failure with reduced ejection fraction.

Kaplan-Meier curves for the occurrence of PEP in patients with and without AF are presented in Figure 1. Patients with AF had a worse prognosis than patients without AF (log-rank test *P* < .001).

### Proteins associated with AF

**Figure 2:**
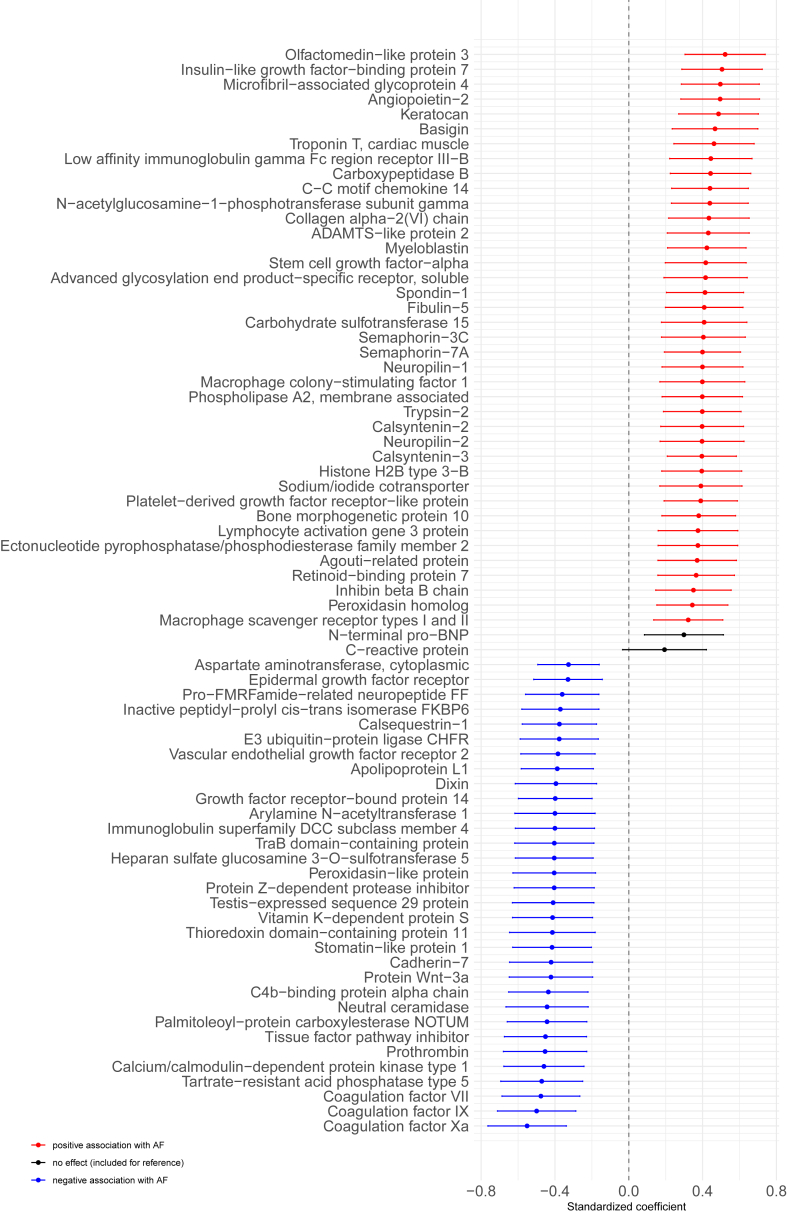
Proteins associated with AF. Coefficients plot of the association between AF and baseline protein measurements, adjusted for age, sex, and kidney function (eGFR [CKD-EPI]). The point denotes the estimated coefficient, and the bar the 95% confidence interval. NT-proBNP and CRP are included for reference. AF = atrial fibrillation; CKD-EPI = Chronic Kidney Disease Epidemiology Collaboration; CRP = C-reactive protein; eGFR = estimated glomerular filtration rate; NT-proBNP = N-terminal pro–B-type natriuretic peptide.

The estimated effect sizes of the proteins associated with AF are presented in Figure 2 and Supplemental Table 2. In total, 71 proteins were significantly associated with AF. Of the 71 proteins, 39 were positively and 32 negatively associated. The top 5 positively associated proteins were olfactomedin-like protein 3 (OLFML3), insulin-like growth factor-binding protein 7 (IGFBP7), microfibril-associated glycoprotein 4 (MFAP4), angiopoietin 2 (ANGPT2), and keratocan (KERA), whereas IGFBP7,^18^ MFAP4,^19^ and ANGPT2^20^ have been related to AF in the past. There has not been much research relating OLFML3 or KERA to AF, although protein-protein interactions in the STRING database can link them to MFAP4, as illustrated in Supplemental Figure 1. The top 5 negatively associated proteins were coagulation factors Xa (F10), IX (F9), and VII (F7); tartrate-resistant acid phosphatase type 5; and calcium/calmodulin-dependent protein kinase (CaMK) type 1. F10, F9, and F7 are related to coagulation, whereas acid phosphatase type 5 is associated with cardiac fibrosis.^21^ CaMK type 1 is part of the broader CaMK family, which plays a role in regulating calcium handling within cardiac cells. Furthermore, its dysregulation is associated with AF.^22^

The results of the analysis with additional adjustment for potential confounding patient characteristics and comorbidities (history of diabetes mellitus, hypertension, hypercholesterolemia, and myocardial infarction; NYHA class; body mass index; and smoking status) and medication use (angiotensin-converting enzyme inhibitor, anticoagulants, aspirin, and loop diuretics) are presented in Supplemental Figure 2. All 71 previously identified proteins remained significantly associated with AF (*P* < .05) while adjusting for the potential confounding patient characteristics and comorbidities. However, after additional adjustment for medication use, 8 of the downregulated proteins were not significantly associated with AF anymore (*P* < .05), specifically coagulation factors Xa, IX, VII and II (F10, F9, F7, F2), tissue factor pathway inhibitor (TFPI), c4b-binding protein alpha chain (C4BPA), vitamin K-dependent protein S (PROS1), and protein Z-dependent protease inhibitor (SERPINA10).

### Associated biological pathways

**Figure 3:**
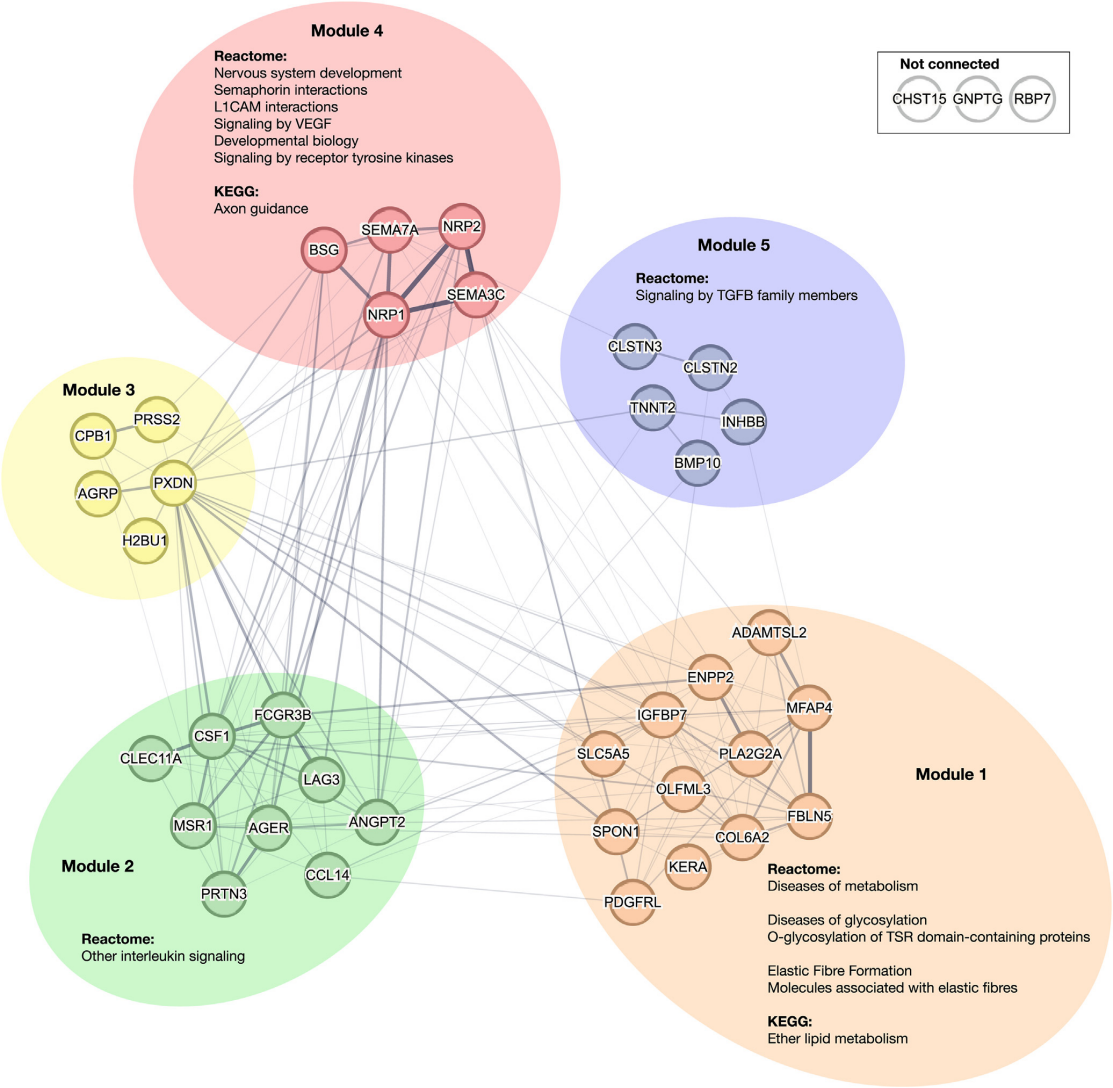
Network of proteins positively associated with AF. Knowledge-based network of proteins positively associated with AF, generated using the STRING database. The width of the edges between proteins represents protein-protein association. The proteins were clustered into 5 protein modules using optimal modularity clustering, as indicated by color. Significantly associated pathways from the KEGG and Reactome pathway databases (FDR < .05) linked with 2 or more proteins are given in text next to the clusters. AF = atrial fibrillation; FDR = false discovery rate; KEGG = Kyoto Encyclopedia of Genes and Genomes.

The knowledge-based network of the 39 proteins positively associated with AF is presented in Figure 3. Optimal modularity clustering resulted in 5 modules that were separately analyzed for association with biological pathways. The modules contained 12, 9, 5, 5, and 5 proteins, respectively, whereas 3 proteins, namely carbohydrate sulfotransferase 15 (CHST15), N-acetylglucosamine-1-phosphate transferase gamma subunit (GNPTG), and retinoid-binding protein 7 (RBP7), had no connections to other proteins in the network. Module 1 was associated with pathways related to elastic fiber formation, ether lipid metabolism, and protein glycosylation; module 2 with interleukin signaling; module 4 with nervous system development; and module 5 with transforming growth factor-beta (TGF-β) signaling. There were no pathways significantly associated with module 3. Detailed distributions of the proteins related to these mechanisms in patients with and without AF are presented in Supplemental Figure 3.

The same analysis was performed for the 32 proteins negatively associated with AF, as illustrated in Supplemental Figure 4. Here, 2 modules were found of 12 and 8 proteins, respectively. The remaining proteins had no connection to any other protein in the network. The first module was associated with WNT- and EGFR signaling, and the second module with pathways related to coagulation. A full table of the associated pathways is presented in Supplemental Table 3.

## Discussion

In this study, we described differences in circulating proteins with putative pathophysiological effects in the CV system in patients with HFrEF with and without AF. We identified biomarkers associated with AF from a panel of 4210 circulating proteins and subsequently examined known and alleged biological pathways related to these biomarkers.

Patients with AF had distinct proteomic profiles in our study compared with patients without AF. The presence of AF was significantly associated with 71 of the 4210 proteins measured. This set of proteins contained well-known and lesser-known proteins described in AF literature. Notable proteins associated with AF included high-sensitivity troponin T,^23^ IGFBP7,^18^ MFAP4,^19^ bone morphogenetic protein 10 (BMP10),^20^^,^^24^ and ANGPT2.^20^ Interestingly, although NT-proBNP was positively associated with AF, this association lost statistical significance after correction for multiple testing (*P* = .006; FDR = .141). The top hit, OLFML3, has not yet been studied in the context of AF. However, as a secreted glycoprotein detectable in the heart and liver, regulated by TGF-β signaling, it would be an interesting target for further study.^25^^,^^26^ Furthermore, knowledge-based protein-protein interaction networks can link it to MFAP4, which may point toward a common mechanism.

Similar to previous studies, patients with AF had more severe HF symptoms, as signified by their higher NYHA class, and had worse HF prognosis.^2^ Furthermore, the duration of HF at baseline was longer for patients with AF. This was expected when considering that exposure to HF can induce AF, and thus, patients who developed HF earlier will have more time to develop AF. We corrected for this in our analyses. Furthermore, a sensitivity analysis with additional correction for NYHA class was conducted, which did not significantly alter the associations of the identified proteins.

Our enrichment analysis revealed several distinct pathways related to the proteins positively associated with AF. The pathway terms of KEGG and Reactome were mainly related to 6 broader categories, as illustrated in further detail in Supplemental Figure 5: elastic fiber formation, protein glycosylation, ether lipid metabolism, nervous system development, interleukin signaling, and TGF-β signaling. AF can structurally alter the heart by increasing extracellular matrix formation and deposition of fibrous material and is closely related to atrial fibrosis.^27^ Therefore, it is not surprising that we find 3 upregulated proteins that are linked to elastic fiber formation (MFAP4, fibulin 5 (FBLN5), and BMP10) in the KEGG and Reactome databases and 7 proteins that are linked to extracellular matrix organization (in addition to the aforementioned, collagen type VI alpha 2 chain (COL6A2), serine protease 2 (PRSS2), peroxidasin (PXDN), and basigin (BSG)). Second, 3 upregulated proteins were linked to protein glycosylation: KERA, spondin 1 (SPON1), and ADAMTS-like 2 (ADAMTSL2). Protein glycosylation is an enzyme-based process in which carbohydrates are attached to proteins. Subtypes of glycosylation can be associated with different AF-related processes. O-glycosylation, more specifically O-GlcNAcylation of the CaMK type 2 enzyme, has been associated with AF by affecting the handling of Ca^2+^ in cardiomyocytes.^28^ In addition, research has shown that patients with AF exhibit unique N-glycosylation patterns, which could signify increased inflammatory response.^29^ Furthermore, protein glycosylation can be associated with neurodegeneration and Alzheimer’s disease, which could affect the development of AF.^30^ Third, 2 upregulated proteins were linked to ether lipid metabolism: ectonucleotide pyrophosphatase/phosphodiesterase family member 2 (autotaxin) and phospholipase A2 group IIA (PLA2G2A). Ether lipids are known to regulate ion channels, whereas ion channel dysfunction is associated with AF.^31^ AF can change the expression or regulation of ion channels, which can result in the shortening of atrial refractoriness and prolongation of atrial action potential.^27^ Furthermore, ether lipids are also associated with neurodegeneration.^31^ Fourth, the autonomic nervous system is known to regulate atrial function and can be an important factor in the development of new-onset AF.^32^ We find 4 proteins linked to (autonomic) nervous system development: semaphorin 7A (SEMA7A) and 3C (SEMA3C) and neuropilin 1 (NRP1) and 2 (NRP2). Fifth, interleukin signaling plays an important role in inflammation and can subsequently be linked with AF, as discussed before.^29^ It is linked with proteinase 3 and colony-stimulating factor 1 (CSF1). Finally, TGF-β signaling can be associated with cardiac fibrosis and remodeling and is linked with BMP10 and inhibin beta B (INHBB).^33^ The variability in these mechanisms and the distribution of their related proteins in Supplemental Figure 3 highlight the heterogeneity of AF and may indicate that the described mechanisms may not be at play for all patients with AF-HFrEF. Therefore, identifying subphenotypes of AF-HFrEF may be an interesting avenue of future research.

Negatively associated proteins were related to coagulation and WNT- and EGFR signaling. Although these pathways can play an important role in AF, these results should be interpreted with caution, given that these pathways can be influenced by differences in anticoagulant use between patients with and without AF.^34^ This is further illustrated by the results of the sensitivity analysis displayed in Supplemental Figure 2.

Our study is the first to use such an extensive proteomic panel to investigate AF in HFrEF. So far, only 1 study has investigated differences in circulating proteins by multiplex panels between patients with HF with and without AF. Santema et al^35^ conducted an investigation of a similar nature, studying the associations of a smaller set of 92 proteins with AF in a cohort of 1620 patients with HF. They found 8 proteins and related them to amyloid-beta based on the implication of SPON1, IGFBP1, and IGFBP7. Although this exact pathway did not emerge from our analyses, we did find associations for SPON1 and IGFBP7 and similar Alzheimer’s disease-related pathways in protein glycosylation and ether lipid metabolism, confirming the relevance of these pathways.^30^^,^^31^ Furthermore, we extend the findings of Santema et al with the current investigation, because our study provides a more comprehensive overview of the pathophysiological pathways at play in patients with HFrEF with AF, by using a wider panel of 4210 proteins.

Underlying mechanisms of AF have been examined in general populations using large-scale genome-wide association studies, which implicated genes associated with cardiac developmental, electrophysiological, contractile, and structural pathways.^36^^,^^37^ In the current study, we find that AF in a cohort of patients with HFrEF is associated with both similar (eg, elastic fiber formation) and different types of pathways (eg, ether lipid metabolism and protein glycosylation). Studies on incident AF in general populations, which used large-scale proteomic approaches, found similar results. Studies in the UK Biobank and the AGES-Reykjavik cohort using 2923 and 4137 serum proteins, respectively, found 172 and 76 proteins, respectively, related to incident AF, many of which are related to ion channel dysfunction and structural remodeling.^38^^,^^39^ Various proteins in these sets display overlap with our results, for example, those for IGFBP7, ANGPT2, and SPON1; however, other findings, such as a strong association between NT-proBNP and incident AF, are not reflected in the results from the current investigation reporting on prevalent AF in patients with HFrEF. Although these differences between commonalities might suggest that HF can modulate proteomic profiles associated with AF, they may also be caused by demographic factors or differences between incident and prevalent AF.

Enhanced knowledge of the pathways and proteins involved in AF in patients with HFrEF has several clinical and scientific implications. Our findings can inspire more personalized management strategies for AF in HFrEF in the long term. These may include the utilization of the identified biomarkers to guide the optimal timing of therapeutic interventions or to inform the selection of specific treatments. Furthermore, our results may provide leads for the identification of proteins that could serve as novel therapeutic targets in drug development. To advance toward such personalized AF-HFrEF management, further research is essential to validate our results and to investigate the clinical utility of the implicated proteomic biomarkers.

Limitations of this study include the relatively small fractions of women (27%), patients with non-Caucasian ethnicity (7%), and patients with NYHA class III/IV (27.8%). The method of measuring the proteins also warrants some consideration. SOMAmer reagents were selected against proteins in their native folded conformations. Therefore, unfolded or denatured proteins are not detected. Furthermore, the proteins in the SomaScan assay are measured in normalized relative fluorescent units rather than absolute concentrations. These are suitable for comparing patients and changes over time within a patient. However, they are not recommended to be used to inform clinical decisions that require absolute concentrations. Different pathways may be involved when AF causes HF to develop vs when HF causes AF to develop. Owing to the setup of the study, we were unable to assess this difference. Although we adjusted our primary analyses for age, sex, kidney function, and HF duration, we chose not to further adjust for differences in the prevalence of comorbid conditions and cardiac risk factors between investigated groups in the main analysis, given that a varying degree of involvement of noncardiac mechanisms can be seen as inherent to the 2 groups. Furthermore, we did not adjust for medication owing to high collinearity between medication use and AF status and subsequent loss of power. However, our sensitivity analysis shows that the results of the presented upregulated proteins are robust to additional adjustment for both these potential confounding factors and medication use. Finally, HFrEF cohorts with extensive measurements of the circulating proteome are scarce, and the current investigation does not provide external validation of the results. Therefore, the identified distinct proteomic profiles and associated biological mechanisms presented in this work should be considered preliminary and warrant confirmation in other cohorts to ensure generalizability and clinical applicability.

## Conclusion

Systemic pathophysiological differences between HFrEF with and without concomitant AF seem to be reflected by circulating proteomic profiles. Differently expressed proteins were related to elastic fiber formation, ether lipid metabolism, protein glycosylation, and nervous system development, all of which are processes that are linked to AF. Our results confirm previous (pre-)clinical findings on AF-related proteins, provide further insights into the biological pathways associated with AF in HF, and could inform future research on novel treatment targets and HF-AF management after validation in other studies.
