# Supplementary material for: Proteomic biomarkers and biological pathways associated with atrial fibrillation in heart failure patients with reduced ejection fraction
Source: Heart Rhythm O2. 2025 Jun 16;6(9):1296–305. doi: 10.1016/j.hroo.2025.06.007 (PMC12635735; doi:10.1016/j.hroo.2025.06.007)
Supplement: Supplementary file 1 — Supplementary Data [file mmc1.docx]

Supplemental Material:

Proteomic biomarkers and biological pathways associated with atrial fibrillation in heart failure patients with reduced ejection fraction

Teun B. Petersen, MSc^a,b,c^; Mylène Barry-Loncq de Jong, MD^a^; Jie Fen Chin, MD^a,d^; Navin Suthahar, MD, PhD^a^; Peter J. van der Spek, PhD^e^; Peter D. Katsikis, MD, PhD^f^; K. Martijn Akkerhuis, MD, PhD^a^; Victor A. Umans, MD, PhD^g^; Rudolf A. de Boer, MD, PhD^a^; Bas M. van Dalen, MD, PhD^a,d^; Jasper J. Brugts, MD, PhD^a^; Folkert W. Asselbergs, MD, PhD^h^; Eric Boersma, PhD^a^; Dimitris Rizopoulos, PhD^b,c^; Sing-Chien Yap, MD, PhD^a^; Isabella Kardys, MD, PhD^a^

a Department of Cardiology, Thorax Center, Cardiovascular Institute, Erasmus MC, Rotterdam, the Netherlands.

b Department of Biostatistics, Erasmus MC, Rotterdam, the Netherlands.

c Department of Epidemiology, Erasmus MC, Rotterdam, the Netherlands.

d Department of Cardiology, Franciscus Gasthuis & Vlietland, Rotterdam, The Netherlands

e Department of Pathology & Clinical Bioinformatics, Erasmus MC, Rotterdam, the Netherlands.
f Department of Immunology, Erasmus MC, Rotterdam, the Netherlands.
g Department of Cardiology, Northwest Clinics, Alkmaar, the Netherlands.
h Department of Cardiology, Amsterdam University Medical Centers, University of Amsterdam, Amsterdam, the Netherlands

**Supplemental methods**

**Proteomic measurements**

Plasma protein concentration levels were measured using the aptamer-based proteomic SomaScan® platform.^1^ SomaScan makes use of single stranded DNA-based protein affinity reagents called SOMAmers® (Slow Off-rate Modified Aptamers). These SOMAmers bind to proteins with high specificity and affinity, and slow dissociation rates, minimizing nonspecific binding interactions. The SomaScanassay's readout is given in normalized relative fluorescent units (RFUs), which are directly proportional to the amount of target protein in the initial sample. Previous studies have reported high assay reproducibility and low technical variability of SomaScan.^2, 3^

The standard processes for normalization, calibration, and quality control (QC) were followed as previously described in Williams et al.^4^ The following normalization and calibration factors were considered acceptable: hybridization control, intraplate median signal normalization and plate scale factors were expected to be between 0.4 and 2.5; the distribution of QC sample ratios was expected to fall for 85% of individual SOMAmer reagents in the total array between 0.8 and 1.2. SOMAmers outside these ranges were omitted in this study. Moreover, SOMAmers with non-human and/or not-validated targets were excluded from further analyses. Whenever multiple SOMAmer versions were present, those with the highest binding affinity were used. This selection resulted in 4210 out of the total 5284 modified aptamers being used in the current analyses. Individual sample quality was evaluated by comparing normalized median signal relative to the external reference standard, with an acceptable normalization scaling range of 0.4 – 2.5. In total, 1066 samples passed the quality-control criteria. Analytic performance of the Somalogic panel is provided in Supplemental Table 1.

**Sample size estimation**

Sample size calculations for the Bio-SHiFT study (total n=398) were based on the expected associations between repeated circulating protein biomarkers and the primary endpoint. In the investigation comprising 382 HFrEF patients, 114 reached the primary end point. For baseline measurements, these numbers are sufficient to detect odds ratios around 2 for the upper quintile of a biomarker associated with the end point (α error .05, power of 80%) when comparing incident cases with non-cases. For repeated measurements, power is further enhanced. Based on input parameters derived from the benchmark blood biomarker NT-proBNP, and using 500 simulations, we calculated that using 3 measurements per person, a difference in change of NT-proBNP level over time of 10 pmol/L per month can be demonstrated between cases and non-cases (Bonferroni corrected α-error: (0.05/4210), power: 87%). This difference is very small in clinical terms, demonstrating that the study has high statistical power.

**Supplemental Tables**

**Supplemental Table 1: Analytic performance of the SOMAscan assay**

| **Attribute** | **Metric** | **Criterion** | **Result** |
| --- | --- | --- | --- |
| Precision (Total CV) | Median %  % in Tail (above 90^th^ %) | ≤7.5%  <15% | 5.0%  11.8% |
| Accuracy | Median QC ratio  % in Tail | N/A  <15% | 1.0  2.4% |
| Signal to Noise | Median | ≥2.5 | 10.5 |
| Limit of Detection (LoD) | Median | ≤100 fM | 90fM |
| Spike and recovery | 25^th^-75^th^ percentile | N/A | 85%-121% |
| Median dynamic range per SOMAmer reagent buffer | Range | N/A | 4.7 logs |
| Interfering Substances | Non-Interfering | Pass | Pass |

**Supplemental Table 2: Proteins associated with AF**

| **ID** | **Gene** | **Name** | **Beta [95%CI]** | **p-value** | **FDR** |
| --- | --- | --- | --- | --- | --- |
| Q9NRN5 | OLFML3 | Olfactomedin-like protein 3 | 0.52 [0.30, 0.74] | <0.001 | 0.005 |
| Q16270 | IGFBP7 | Insulin-like growth factor-binding protein 7 | 0.51 [0.29, 0.72] | <0.001 | 0.005 |
| P55083 | MFAP4 | Microfibril-associated glycoprotein 4 | 0.50 [0.28, 0.71] | <0.001 | 0.005 |
| O15123 | ANGPT2 | Angiopoietin-2 | 0.50 [0.28, 0.71] | <0.001 | 0.005 |
| O60938 | KERA | Keratocan | 0.49 [0.27, 0.70] | <0.001 | 0.007 |
| P35613 | BSG | Basigin | 0.47 [0.24, 0.70] | <0.001 | 0.019 |
| P45379 | TNNT2 | Troponin T, cardiac muscle | 0.46 [0.24, 0.68] | <0.001 | 0.012 |
| O75015 | FCGR3B | Low affinity immunoglobulin gamma Fc region receptor III-B | 0.44 [0.22, 0.67] | <0.001 | 0.019 |
| P15086 | CPB1 | Carboxypeptidase B | 0.44 [0.23, 0.66] | <0.001 | 0.019 |
| Q16627 | CCL14 | C-C motif chemokine 14 | 0.44 [0.23, 0.65] | <0.001 | 0.012 |
| Q9UJJ9 | GNPTG | N-acetylglucosamine-1-phosphotransferase subunit gamma | 0.44 [0.23, 0.65] | <0.001 | 0.012 |
| P12110 | COL6A2 | Collagen alpha-2(VI) chain | 0.43 [0.22, 0.65] | <0.001 | 0.019 |
| Q86TH1 | ADAMTSL2 | ADAMTS-like protein 2 | 0.43 [0.21, 0.65] | <0.001 | 0.021 |
| P24158 | PRTN3 | Myeloblastin | 0.42 [0.21, 0.64] | <0.001 | 0.019 |
| Q9Y240 | CLEC11A | Stem cell growth factor-alpha | 0.42 [0.20, 0.64] | <0.001 | 0.024 |
| Q15109 | AGER | Advanced glycosylation end product-specific receptor, soluble | 0.42 [0.19, 0.64] | <0.001 | 0.030 |
| Q9HCB6 | SPON1 | Spondin-1 | 0.41 [0.20, 0.62] | <0.001 | 0.019 |
| Q9UBX5 | FBLN5 | Fibulin-5 | 0.41 [0.20, 0.62] | <0.001 | 0.021 |
| Q7LFX5 | CHST15 | Carbohydrate sulfotransferase 15 | 0.41 [0.18, 0.64] | <0.001 | 0.040 |
| Q99985 | SEMA3C | Semaphorin-3C | 0.40 [0.18, 0.63] | <0.001 | 0.038 |
| O75326 | SEMA7A | Semaphorin-7A | 0.40 [0.19, 0.61] | <0.001 | 0.022 |
| O14786 | NRP1 | Neuropilin-1 | 0.40 [0.18, 0.62] | <0.001 | 0.032 |
| P09603 | CSF1 | Macrophage colony-stimulating factor 1 | 0.40 [0.17, 0.63] | <0.001 | 0.045 |
| P14555 | PLA2G2A | Phospholipase A2, membrane associated | 0.40 [0.18, 0.62] | <0.001 | 0.032 |
| P07478 | PRSS2 | Trypsin-2 | 0.40 [0.19, 0.61] | <0.001 | 0.025 |
| Q9H4D0 | CLSTN2 | Calsyntenin-2 | 0.40 [0.17, 0.62] | <0.001 | 0.038 |
| O60462 | NRP2 | Neuropilin-2 | 0.40 [0.17, 0.62] | <0.001 | 0.043 |
| Q9BQT9 | CLSTN3 | Calsyntenin-3 | 0.40 [0.21, 0.58] | <0.001 | 0.012 |
| Q8N257 | HIST3H2BB | Histone H2B type 3-B | 0.40 [0.18, 0.61] | <0.001 | 0.032 |
| Q92911 | SLC5A5 | Sodium/iodide cotransporter | 0.39 [0.17, 0.61] | <0.001 | 0.041 |
| Q15198 | PDGFRL | Platelet-derived growth factor receptor-like protein | 0.39 [0.19, 0.59] | <0.001 | 0.020 |
| O95393 | BMP10 | Bone morphogenetic protein 10 | 0.38 [0.18, 0.58] | <0.001 | 0.025 |
| P18627 | LAG3 | Lymphocyte activation gene 3 protein | 0.37 [0.16, 0.59] | <0.001 | 0.044 |
| Q13822 | ENPP2 | Ectonucleotide pyrophosphatase/phosphodiesterase family member 2 | 0.37 [0.16, 0.59] | <0.001 | 0.044 |
| O00253 | AGRP | Agouti-related protein | 0.37 [0.16, 0.58] | <0.001 | 0.043 |
| Q96R05 | RBP7 | Retinoid-binding protein 7 | 0.37 [0.16, 0.57] | <0.001 | 0.041 |
| P09529 | INHBB | Inhibin beta B chain | 0.35 [0.15, 0.55] | <0.001 | 0.049 |
| Q92626 | PXDN | Peroxidasin homolog | 0.34 [0.15, 0.54] | <0.001 | 0.037 |
| P21757 | MSR1 | Macrophage scavenger receptor types I and II | 0.32 [0.14, 0.51] | <0.001 | 0.048 |
| P16860 | NPPB | N-terminal pro-BNP | 0.30 [0.09, 0.51] | 0.006 | 0.141 |
| P02741 | CRP | C-reactive protein | 0.19 [-0.03, 0.42] | 0.094 | 0.502 |
| P17174 | GOT1 | Aspartate aminotransferase, cytoplasmic | -0.33 [-0.49, -0.16] | <0.001 | 0.020 |
| P00533 | EGFR | Epidermal growth factor receptor | -0.33 [-0.52, -0.14] | <0.001 | 0.038 |
| O15130 | NPFF | Pro-FMRFamide-related neuropeptide FF | -0.36 [-0.56, -0.16] | <0.001 | 0.033 |
| O75344 | FKBP6 | Inactive peptidyl-prolyl cis-trans isomerase FKBP6 | -0.37 [-0.58, -0.16] | <0.001 | 0.038 |
| P31415 | CASQ1 | Calsequestrin-1 | -0.38 [-0.58, -0.17] | <0.001 | 0.027 |
| Q96EP1 | CHFR | E3 ubiquitin-protein ligase CHFR | -0.38 [-0.59, -0.17] | <0.001 | 0.038 |
| P35968 | KDR | Vascular endothelial growth factor receptor 2 | -0.38 [-0.58, -0.18] | <0.001 | 0.024 |
| O14791 | APOL1 | Apolipoprotein L1 | -0.39 [-0.58, -0.19] | <0.001 | 0.019 |
| Q155Q3 | DIXDC1 | Dixin | -0.39 [-0.61, -0.17] | <0.001 | 0.037 |
| Q14449 | GRB14 | Growth factor receptor-bound protein 14 | -0.40 [-0.60, -0.20] | <0.001 | 0.019 |
| P18440 | NAT1 | Arylamine N-acetyltransferase 1 | -0.40 [-0.62, -0.18] | <0.001 | 0.030 |
| Q8TDY8 | IGDCC4 | Immunoglobulin superfamily DCC subclass member 4 | -0.40 [-0.61, -0.19] | <0.001 | 0.027 |
| Q9H4I3 | TRABD | TraB domain-containing protein | -0.40 [-0.62, -0.19] | <0.001 | 0.026 |
| Q8IZT8 | HS3ST5 | Heparan sulfate glucosamine 3-O-sulfotransferase 5 | -0.40 [-0.61, -0.19] | <0.001 | 0.024 |
| A1KZ92 | PXDNL | Peroxidasin-like protein | -0.40 [-0.63, -0.18] | <0.001 | 0.035 |
| Q9UK55 | SERPINA10 | Protein Z-dependent protease inhibitor | -0.40 [-0.62, -0.19] | <0.001 | 0.027 |
| Q8N6K0 | TEX29 | Testis-expressed sequence 29 protein | -0.41 [-0.63, -0.19] | <0.001 | 0.027 |
| P07225 | PROS1 | Vitamin K-dependent protein S | -0.41 [-0.63, -0.20] | <0.001 | 0.024 |
| Q6PKC3 | TXNDC11 | Thioredoxin domain-containing protein 11 | -0.41 [-0.65, -0.18] | <0.001 | 0.038 |
| Q9UBI4 | STOML1 | Stomatin-like protein 1 | -0.42 [-0.63, -0.20] | <0.001 | 0.021 |
| Q9ULB5 | CDH7 | Cadherin-7 | -0.42 [-0.65, -0.20] | <0.001 | 0.027 |
| P56704 | WNT3A | Protein Wnt-3a | -0.42 [-0.65, -0.20] | <0.001 | 0.027 |
| P04003 | C4BPA | C4b-binding protein alpha chain | -0.44 [-0.65, -0.22] | <0.001 | 0.019 |
| Q9NR71 | ASAH2 | Neutral ceramidase | -0.44 [-0.67, -0.22] | <0.001 | 0.019 |
| Q6P988 | NOTUM | Palmitoleoyl-protein carboxylesterase NOTUM | -0.44 [-0.66, -0.23] | <0.001 | 0.019 |
| P10646 | TFPI | Tissue factor pathway inhibitor | -0.45 [-0.67, -0.23] | <0.001 | 0.019 |
| P00734 | F2 | Prothrombin | -0.45 [-0.68, -0.23] | <0.001 | 0.019 |
| Q14012 | CAMK1 | Calcium/calmodulin-dependent protein kinase type 1 | -0.46 [-0.68, -0.24] | <0.001 | 0.012 |
| P13686 | ACP5 | Tartrate-resistant acid phosphatase type 5 | -0.47 [-0.69, -0.25] | <0.001 | 0.012 |
| P08709 | F7 | Coagulation factor VII | -0.48 [-0.69, -0.27] | <0.001 | 0.007 |
| P00740 | F9 | Coagulation factor IX | -0.50 [-0.71, -0.29] | <0.001 | 0.005 |
| P00742 | F10 | Coagulation factor Xa | -0.55 [-0.76, -0.34] | <0.001 | 0.002 |

**Supplemental Table 3: Associated KEGG and Reactome terms per protein module**

|  |  | **Positively associated protein modules** | | | | | | **Negatively associated protein modules** | | |
| --- | --- | --- | --- | --- | --- | --- | --- | --- | --- | --- |
| ID | Pathway name | 1 | 2 | 3 | 4 | 5 | 1 | | 2 |  |
| M2130 | KEGG_ETHER_LIPID_METABOLISM | **2.4E-02** | 1.0E+00 | 1.0E+00 | 1.0E+00 | 1.0E+00 | 1.0E+00 | | 1.0E+00 |  |
| M27275 | REACTOME_DISEASES_OF_GLYCOSYLATION | **2.4E-02** | 1.0E+00 | 1.0E+00 | 1.0E+00 | 1.0E+00 | 1.0E+00 | | 1.0E+00 |  |
| M27134 | REACTOME_MOLECULES_ASSOCIATED_WITH_ELASTIC_FIBRES | **2.4E-02** | 1.0E+00 | 1.0E+00 | 1.0E+00 | **3.1E-02** | 1.0E+00 | | 1.0E+00 |  |
| M27417 | REACTOME_O_GLYCOSYLATION_OF_TSR_DOMAIN_CONTAINING_PROTEINS | **2.4E-02** | 1.0E+00 | 1.0E+00 | 1.0E+00 | 1.0E+00 | 1.0E+00 | | 1.0E+00 |  |
| M26969 | REACTOME_ELASTIC_FIBRE_FORMATION | **2.9E-02** | 1.0E+00 | 1.0E+00 | 1.0E+00 | **3.1E-02** | 1.0E+00 | | 1.0E+00 |  |
| M27554 | REACTOME_DISEASES_OF_METABOLISM | **3.7E-02** | 1.0E+00 | 1.0E+00 | 1.0E+00 | 1.0E+00 | 1.0E+00 | | 1.0E+00 |  |
| M27303 | REACTOME_DISEASES_ASSOCIATED_WITH_O_GLYCOSYLATION_OF_PROTEINS | **4.4E-02** | 1.0E+00 | 1.0E+00 | 1.0E+00 | 1.0E+00 | 1.0E+00 | | 1.0E+00 |  |
| M27385 | REACTOME_OTHER_INTERLEUKIN_SIGNALING | 1.0E+00 | **3.8E-02** | 1.0E+00 | 1.0E+00 | 1.0E+00 | 1.0E+00 | | 1.0E+00 |  |
| M5539 | KEGG_AXON_GUIDANCE | 1.0E+00 | 1.0E+00 | 1.0E+00 | **4.3E-03** | 1.0E+00 | 1.0E+00 | | 1.0E+00 |  |
| M7923 | REACTOME_SEMAPHORIN_INTERACTIONS | 1.0E+00 | 1.0E+00 | 1.0E+00 | **9.4E-03** | 1.0E+00 | 1.0E+00 | | 1.0E+00 |  |
| M872 | REACTOME_L1CAM_INTERACTIONS | 1.0E+00 | 1.0E+00 | 1.0E+00 | **1.7E-02** | 1.0E+00 | 1.7E-01 | | 1.0E+00 |  |
| M27077 | REACTOME_SIGNALING_BY_VEGF | 1.0E+00 | 1.0E+00 | 1.0E+00 | **1.7E-02** | 1.0E+00 | 1.7E-01 | | 1.0E+00 |  |
| M29853 | REACTOME_NERVOUS_SYSTEM_DEVELOPMENT | 5.2E-01 | 1.0E+00 | 1.0E+00 | **1.8E-02** | 1.0E+00 | 5.0E-01 | | 1.0E+00 |  |
| M27355 | REACTOME_PROTON_COUPLED_MONOCARBOXYLATE_TRANSPORT | 1.0E+00 | 1.0E+00 | 1.0E+00 | **1.8E-02** | 1.0E+00 | 1.0E+00 | | 1.0E+00 |  |
| M27378 | REACTOME_NRCAM_INTERACTIONS | 1.0E+00 | 1.0E+00 | 1.0E+00 | **2.8E-02** | 1.0E+00 | 1.0E+00 | | 1.0E+00 |  |
| M27379 | REACTOME_CHL1_INTERACTIONS | 1.0E+00 | 1.0E+00 | 1.0E+00 | **2.8E-02** | 1.0E+00 | 1.0E+00 | | 1.0E+00 |  |
| M509 | REACTOME_DEVELOPMENTAL_BIOLOGY | 6.9E-01 | 1.0E+00 | 1.0E+00 | **2.8E-02** | 1.0E+00 | 3.1E-01 | | 1.0E+00 |  |
| M27334 | REACTOME_TRANSPORT_OF_BILE_SALTS_AND_ORGANIC_ACIDS_METAL_IONS_AND_AMINE_COMPOUNDS | 1.0E+00 | 1.0E+00 | 1.0E+00 | **2.8E-02** | 1.0E+00 | 1.0E+00 | | 1.0E+00 |  |
| M7578 | REACTOME_SEMA3A_PLEXIN_REPULSION_SIGNALING_BY_INHIBITING_INTEGRIN_ADHESION | 1.0E+00 | 1.0E+00 | 1.0E+00 | **2.8E-02** | 1.0E+00 | 1.0E+00 | | 1.0E+00 |  |
| M16498 | REACTOME_SEMA3A_PAK_DEPENDENT_AXON_REPULSION | 1.0E+00 | 1.0E+00 | 1.0E+00 | **2.8E-02** | 1.0E+00 | 1.0E+00 | | 1.0E+00 |  |
| M8245 | REACTOME_CRMPS_IN_SEMA3A_SIGNALING | 1.0E+00 | 1.0E+00 | 1.0E+00 | **2.8E-02** | 1.0E+00 | 1.0E+00 | | 1.0E+00 |  |
| M27474 | REACTOME_SLC_TRANSPORTER_DISORDERS | 8.0E-02 | 1.0E+00 | 1.0E+00 | **3.1E-02** | 1.0E+00 | 1.0E+00 | | 1.0E+00 |  |
| M4974 | REACTOME_BASIGIN_INTERACTIONS | 1.0E+00 | 1.0E+00 | 1.0E+00 | **3.3E-02** | 1.0E+00 | 1.0E+00 | | 1.0E+00 |  |
| M45014 | REACTOME_ASPIRIN_ADME | 1.0E+00 | 1.0E+00 | 1.0E+00 | **3.4E-02** | 1.0E+00 | 1.0E+00 | | 1.0E+00 |  |
| M41729 | REACTOME_ATTACHMENT_AND_ENTRY | 1.0E+00 | 1.0E+00 | 1.0E+00 | **3.7E-02** | 1.0E+00 | 1.0E+00 | | 1.0E+00 |  |
| M17157 | REACTOME_PYRUVATE_METABOLISM | 1.0E+00 | 1.0E+00 | 1.0E+00 | **3.7E-02** | 1.0E+00 | 1.0E+00 | | 1.0E+00 |  |
| M10959 | REACTOME_OTHER_SEMAPHORIN_INTERACTIONS | 1.0E+00 | 1.0E+00 | 1.0E+00 | **4.3E-02** | 1.0E+00 | 1.0E+00 | | 1.0E+00 |  |
| M490 | REACTOME_PYRUVATE_METABOLISM_AND_CITRIC_ACID_TCA_CYCLE | 1.0E+00 | 1.0E+00 | 1.0E+00 | **4.3E-02** | 1.0E+00 | 1.0E+00 | | 1.0E+00 |  |
| M5988 | REACTOME_SLC_MEDIATED_TRANSMEMBRANE_TRANSPORT | 1.3E-01 | 1.0E+00 | 1.0E+00 | **4.3E-02** | 1.0E+00 | 1.0E+00 | | 1.0E+00 |  |
| M878 | REACTOME_SIGNAL_TRANSDUCTION_BY_L1 | 1.0E+00 | 1.0E+00 | 1.0E+00 | **4.3E-02** | 1.0E+00 | 9.7E-02 | | 1.0E+00 |  |
| M45028 | REACTOME_EARLY_SARS_COV_2_INFECTION_EVENTS | 1.0E+00 | 1.0E+00 | 1.0E+00 | **4.5E-02** | 1.0E+00 | 1.0E+00 | | 1.0E+00 |  |
| MM15587 | REACTOME_SIGNALING_BY_RECEPTOR_TYROSINE_KINASES | 4.8E-01 | 1.0E+00 | 1.0E+00 | **4.7E-02** | 1.0E+00 | 1.5E-01 | | 1.0E+00 |  |
| M17673 | KEGG_CARDIAC_MUSCLE_CONTRACTION | 1.0E+00 | 1.0E+00 | 1.0E+00 | 1.0E+00 | **3.1E-02** | 1.0E+00 | | 1.0E+00 |  |
| M835 | KEGG_DILATED_CARDIOMYOPATHY | 1.0E+00 | 1.0E+00 | 1.0E+00 | 1.0E+00 | **3.4E-02** | 1.0E+00 | | 1.0E+00 |  |
| M8728 | KEGG_HYPERTROPHIC_CARDIOMYOPATHY_HCM | 1.0E+00 | 1.0E+00 | 1.0E+00 | 1.0E+00 | **3.8E-02** | 1.0E+00 | | 1.0E+00 |  |
| M47821 | KEGG_MEDICUS_REFERENCE_BMP9_10_SIGNALING_PATHWAY | 1.0E+00 | 1.0E+00 | 1.0E+00 | 1.0E+00 | **3.1E-02** | 1.0E+00 | | 1.0E+00 |  |
| M27871 | REACTOME_SIGNALING_BY_TGFB_FAMILY_MEMBERS | 1.0E+00 | 1.0E+00 | 1.0E+00 | 1.0E+00 | **1.8E-02** | 1.0E+00 | | 1.0E+00 |  |
| MM14830 | REACTOME_GLYCOPROTEIN_HORMONES | 1.0E+00 | 1.0E+00 | 1.0E+00 | 1.0E+00 | **3.1E-02** | 1.0E+00 | | 1.0E+00 |  |
| M756 | REACTOME_PEPTIDE_HORMONE_BIOSYNTHESIS | 1.0E+00 | 1.0E+00 | 1.0E+00 | 1.0E+00 | **3.1E-02** | 1.0E+00 | | 1.0E+00 |  |
| M26965 | REACTOME_SIGNALING_BY_ACTIVIN | 1.0E+00 | 1.0E+00 | 1.0E+00 | 1.0E+00 | **3.1E-02** | 1.0E+00 | | 1.0E+00 |  |
| M18647 | REACTOME_STRIATED_MUSCLE_CONTRACTION | 1.0E+00 | 1.0E+00 | 1.0E+00 | 1.0E+00 | **3.1E-02** | 1.0E+00 | | 1.0E+00 |  |
| M1662 | REACTOME_SIGNALING_BY_BMP | 1.0E+00 | 1.0E+00 | 1.0E+00 | 1.0E+00 | **3.1E-02** | 1.0E+00 | | 1.0E+00 |  |
| MM15026 | REACTOME_MUSCLE_CONTRACTION | 1.0E+00 | 1.0E+00 | 1.0E+00 | 1.0E+00 | **4.7E-02** | 1.5E-01 | | 1.0E+00 |  |
| M27211 | REACTOME_PEPTIDE_HORMONE_METABOLISM | 1.0E+00 | 1.0E+00 | 7.1E-02 | 1.0E+00 | **4.7E-02** | 1.0E+00 | | 1.0E+00 |  |
| M47385 | KEGG_MEDICUS_VARIANT_AMPLIFIED_EGFR_TO_PLCG_CAMK_SIGNALING_PATHWAY | 1.0E+00 | 1.0E+00 | 1.0E+00 | 1.0E+00 | 1.0E+00 | **2.8E-02** | | 1.0E+00 |  |
| M47384 | KEGG_MEDICUS_REFERENCE_EGF_EGFR_PLCG_CAMK_SIGNALING_PATHWAY | 1.0E+00 | 1.0E+00 | 1.0E+00 | 1.0E+00 | 1.0E+00 | **2.8E-02** | | 1.0E+00 |  |
| M47834 | KEGG_MEDICUS_REFERENCE_WNT_SIGNALING_MODULATION_WNT_INHIBITOR | 1.0E+00 | 1.0E+00 | 1.0E+00 | 1.0E+00 | 1.0E+00 | **2.8E-02** | | 1.0E+00 |  |
| M16894 | KEGG_COMPLEMENT_AND_COAGULATION_CASCADES | 1.0E+00 | 1.0E+00 | 1.0E+00 | 1.0E+00 | 1.0E+00 | 1.0E+00 | | **1.8E-11** |  |
| M47783 | KEGG_MEDICUS_REFERENCE_LECTIN_PATHWAY_OF_COAGULATION_CASCADE_PROTHROMBIN_TO_THROMBIN | 1.0E+00 | 1.0E+00 | 1.0E+00 | 1.0E+00 | 1.0E+00 | 1.0E+00 | | **2.9E-02** |  |
| MM14613 | REACTOME_GAMMA_CARBOXYLATION_OF_PROTEIN_PRECURSORS | 1.0E+00 | 1.0E+00 | 1.0E+00 | 1.0E+00 | 1.0E+00 | 1.0E+00 | | **1.4E-11** |  |
| MM14614 | REACTOME_TRANSPORT_OF_GAMMA_CARBOXYLATED_PROTEIN_PRECURSORS_FROM_THE_ENDOPLASMIC_RETICULUM_TO_THE_GOLGI_APPARATUS | 1.0E+00 | 1.0E+00 | 1.0E+00 | 1.0E+00 | 1.0E+00 | 1.0E+00 | | **1.8E-11** |  |
| M12484 | REACTOME_GAMMA_CARBOXYLATION_TRANSPORT_AND_AMINO_TERMINAL_CLEAVAGE_OF_PROTEINS | 1.0E+00 | 1.0E+00 | 1.0E+00 | 1.0E+00 | 1.0E+00 | 1.0E+00 | | **2.4E-11** |  |
| M26977 | REACTOME_REMOVAL_OF_AMINOTERMINAL_PROPEPTIDES_FROM_GAMMA_CARBOXYLATED_PROTEINS | 1.0E+00 | 1.0E+00 | 1.0E+00 | 1.0E+00 | 1.0E+00 | 1.0E+00 | | **2.4E-11** |  |
| M2844 | REACTOME_FORMATION_OF_FIBRIN_CLOT_CLOTTING_CASCADE | 1.0E+00 | 1.2E-01 | 1.0E+00 | 1.0E+00 | 1.0E+00 | 1.0E+00 | | **5.0E-11** |  |
| M26948 | REACTOME_EXTRINSIC_PATHWAY_OF_FIBRIN_CLOT_FORMATION | 1.0E+00 | 1.0E+00 | 1.0E+00 | 1.0E+00 | 1.0E+00 | 1.0E+00 | | **4.1E-10** |  |
| M26993 | REACTOME_GAMMA_CARBOXYLATION_HYPUSINYLATION_HYDROXYLATION_AND_ARYLSULFATASE_ACTIVATION | 1.0E+00 | 1.0E+00 | 1.0E+00 | 1.0E+00 | 1.0E+00 | 1.0E+00 | | **2.0E-09** |  |
| M14766 | REACTOME_INTRINSIC_PATHWAY_OF_FIBRIN_CLOT_FORMATION | 1.0E+00 | 1.0E+00 | 1.0E+00 | 1.0E+00 | 1.0E+00 | 1.0E+00 | | **1.2E-07** |  |
| M39000 | REACTOME_DEFECTIVE_FACTOR_VIII_CAUSES_HEMOPHILIA_A | 1.0E+00 | 1.0E+00 | 1.0E+00 | 1.0E+00 | 1.0E+00 | 1.0E+00 | | **6.5E-07** |  |
| M38996 | REACTOME_DEFECTS_OF_CONTACT_ACTIVATION_SYSTEM_CAS_AND_KALLIKREIN_KININ_SYSTEM_KKS | 1.0E+00 | 1.0E+00 | 1.0E+00 | 1.0E+00 | 1.0E+00 | 1.0E+00 | | **5.1E-06** |  |
| M8395 | REACTOME_HEMOSTASIS | 1.0E+00 | 1.5E-01 | 1.0E+00 | 3.4E-01 | 1.0E+00 | 5.6E-01 | | **1.8E-05** |  |
| M17879 | REACTOME_COMMON_PATHWAY_OF_FIBRIN_CLOT_FORMATION | 1.0E+00 | 1.1E-01 | 1.0E+00 | 1.0E+00 | 1.0E+00 | 1.0E+00 | | **1.8E-05** |  |
| M39001 | REACTOME_DEFECTIVE_FACTOR_IX_CAUSES_HEMOPHILIA_B | 1.0E+00 | 1.0E+00 | 1.0E+00 | 1.0E+00 | 1.0E+00 | 1.0E+00 | | **2.2E-04** |  |
| M19806 | REACTOME_POST_TRANSLATIONAL_PROTEIN_MODIFICATION | 1.9E-01 | 2.8E-01 | 1.0E+00 | 1.0E+00 | 1.0E+00 | 7.5E-01 | | **2.8E-04** |  |
| M19752 | REACTOME_COMPLEMENT_CASCADE | 1.0E+00 | 1.0E+00 | 1.0E+00 | 1.0E+00 | 1.0E+00 | 1.0E+00 | | **3.3E-04** |  |
| MM15018 | REACTOME_METABOLISM_OF_PROTEINS | 2.4E-01 | 3.5E-01 | 4.9E-01 | 1.0E+00 | 3.9E-01 | 8.1E-01 | | **6.2E-04** |  |
| M39004 | REACTOME_DEFECTIVE_F9_ACTIVATION | 1.0E+00 | 1.0E+00 | 1.0E+00 | 1.0E+00 | 1.0E+00 | 1.0E+00 | | **2.0E-02** |  |
| M48008 | REACTOME_PROTEIN_HYDROXYLATION | 1.0E+00 | 1.0E+00 | 1.0E+00 | 1.0E+00 | 1.0E+00 | 1.0E+00 | | **2.4E-02** |  |
| M16312 | REACTOME_CELL_SURFACE_INTERACTIONS_AT_THE_VASCULAR_WALL | 1.0E+00 | 1.9E-01 | 1.0E+00 | 1.3E-01 | 1.0E+00 | 2.4E-01 | | **2.9E-02** |  |
| M27285 | REACTOME_REGULATION_OF_INSULIN_LIKE_GROWTH_FACTOR_IGF_TRANSPORT_AND_UPTAKE_BY_INSULIN_LIKE_GROWTH_FACTOR_BINDING_PROTEINS_IGFBPS | 2.7E-01 | 1.9E-01 | 1.0E+00 | 1.0E+00 | 1.0E+00 | 2.4E-01 | | **2.9E-02** |  |
| M861 | REACTOME_THROMBIN_SIGNALLING_THROUGH_PROTEINASE_ACTIVATED_RECEPTORS_PARS | 1.0E+00 | 1.0E+00 | 1.0E+00 | 1.0E+00 | 1.0E+00 | 1.0E+00 | | **2.9E-02** |  |
| M26943 | REACTOME_BMAL1_CLOCK_NPAS2_ACTIVATES_CIRCADIAN_GENE_EXPRESSION | 1.0E+00 | 1.0E+00 | 1.0E+00 | 1.0E+00 | 1.0E+00 | 1.0E+00 | | **2.9E-02** |  |

**Supplemental Figures**

**Supplemental Figure 1: protein-protein interaction network linking top 5 positively associated proteins and their standardized distributions**

**
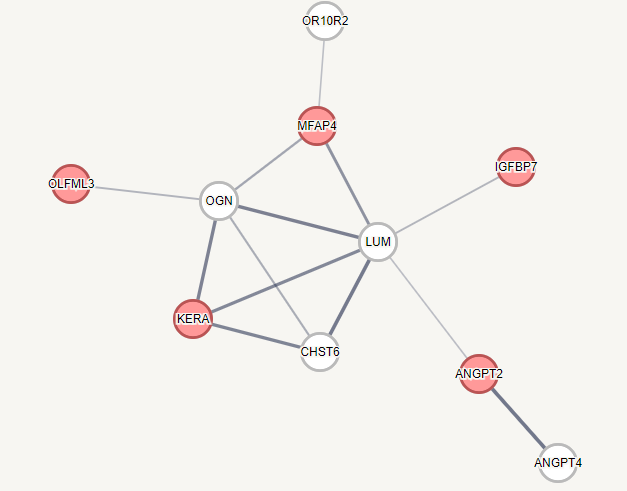
** **
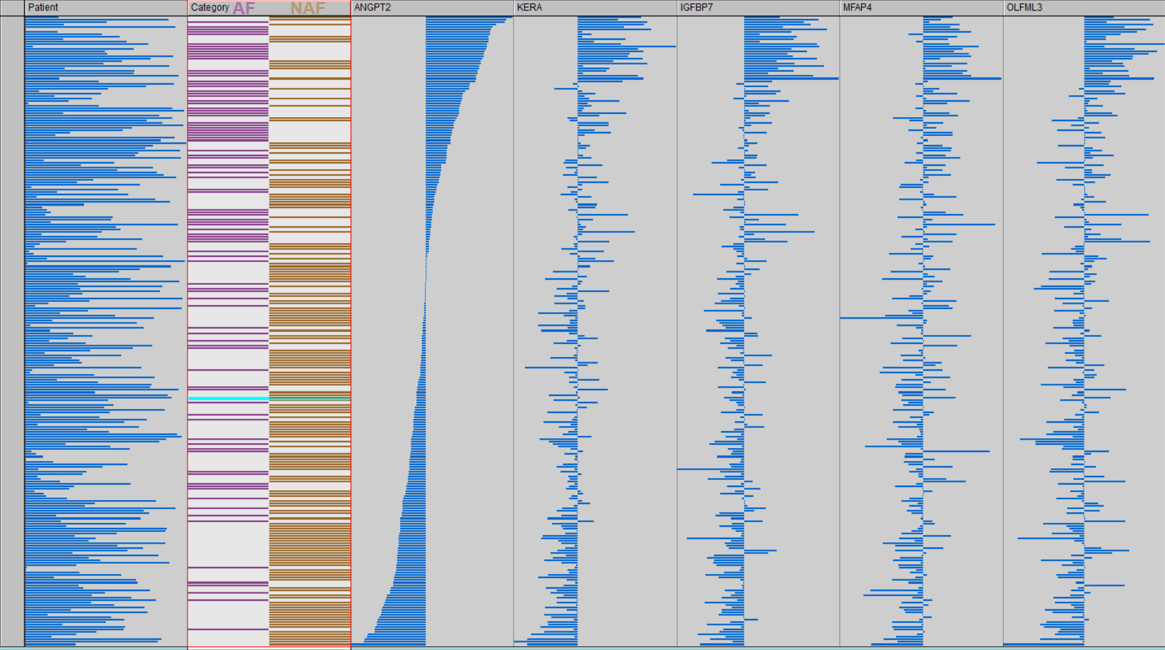
**

**Supplemental Figure 2:**

**Legend:** Coefficients plot of the association between AF and baseline measurements of proteins selected in main analysis (model 1), with three levels of adjustment. The point denotes the estimated coefficient, and the bar the 95% confidence interval. NT-proBNP and CRP are included for reference.

**Supplemental Figure 3: Standardized distribution of described proteins related to biological mechanisms**

**
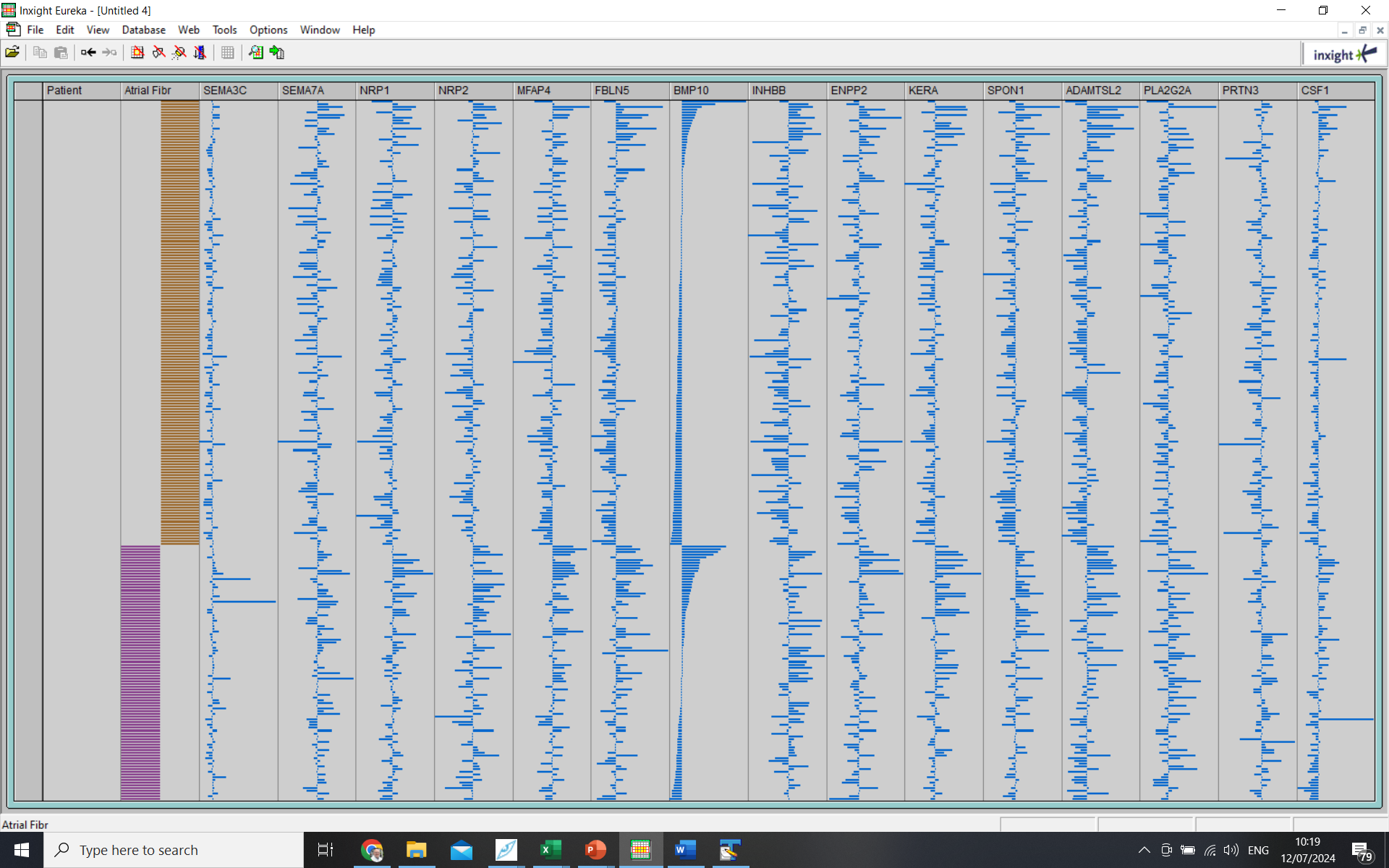
**

**Supplemental Figure 4:** Network of proteins negatively associated with AF

**Legend:** Knowledge-based network of proteins negatively associated with AF, generated using the STRING database. The width of the edges between proteins represents protein-protein association. The proteins were clustered into five protein modules using optimal modularity clustering, as indicated by color. Significantly associated pathways from the KEGG and Reactome pathway databases (FDR>0.05) linked with two or more proteins are given in text next to the clusters.

**Supplemental Figure 5:** Overarching categories for the found AF-related pathways and linked proteins

**Supplemental References**

1. Gold L, Ayers D, Bertino J, Bock C, Bock A, Brody EN, et al. Aptamer-Based Multiplexed Proteomic Technology for Biomarker Discovery. PLoS One. 2010;5(12):e15004.

2. Kim CH, Tworoger SS, Stampfer MJ, Dillon ST, Gu X, Sawyer SJ, et al. Stability and reproducibility of proteomic profiles measured with an aptamer-based platform. Sci Rep. 2018;8(1):1-10.

3. Candia J, Cheung F, Kotliarov Y, Fantoni G, Sellers B, Griesman T, et al. Assessment of variability in the SOMAscan assay. Sci Rep. 2017;7(1):14248.

4. Williams SA, Kivimaki M, Langenberg C, Hingorani AD, Casas JP, Bouchard C, et al. Plasma protein patterns as comprehensive indicators of health. Nature medicine. 2019;25(12):1851-7.
